# Supplementary material for: Resolving the full spectrum of human genome variation using Linked-Reads
Source: Genome Res. 2019 Apr;29(4):635–45. doi: 10.1101/gr.234443.118 (PMC6442396; doi:10.1101/gr.234443.118)
Supplement: Supplemental Material [file supp_gr.234443.118_Supplemental_longranger-2.2.zip › longranger-2.2.tar.bz2!/lr2.2-public/lib/stan/cmdstan/stan/doc/api/html/namespacestan_1_1gm.html]

Stan: stan::gm Namespace Reference


|  |  |
| --- | --- |
|  | Stan  2.5.0  probability, sampling & optimization |


- Main Page
- Related Pages
- Namespaces
- Classes
- Files

- Namespace List
- Namespace Members

All Classes Namespaces Files Functions Variables Typedefs Enumerations Enumerator Friends Macros Pages

- stan
- gm

Classes |
Typedefs |
Functions |
Variables

stan::gm Namespace Reference

Directed graphical model compiler.
More...

|  |  |
| --- | --- |
| Classes | |
| class | arg\_adapt |
|  | |
| class | arg\_adapt\_delta |
|  | |
| class | arg\_adapt\_engaged |
|  | |
| class | arg\_adapt\_gamma |
|  | |
| class | arg\_adapt\_init\_buffer |
|  | |
| class | arg\_adapt\_kappa |
|  | |
| class | arg\_adapt\_t0 |
|  | |
| class | arg\_adapt\_term\_buffer |
|  | |
| class | arg\_adapt\_window |
|  | |
| class | arg\_bfgs |
|  | |
| class | arg\_data |
|  | |
| class | arg\_data\_file |
|  | |
| class | arg\_dense\_e |
|  | |
| class | arg\_diag\_e |
|  | |
| class | arg\_diagnose |
|  | |
| class | arg\_diagnostic\_file |
|  | |
| class | arg\_engine |
|  | |
| class | arg\_fail |
|  | |
| class | arg\_fixed\_param |
|  | |
| class | arg\_history\_size |
|  | |
| class | arg\_hmc |
|  | |
| class | arg\_id |
|  | |
| class | arg\_init |
|  | |
| class | arg\_init\_alpha |
|  | |
| class | arg\_int\_time |
|  | |
| class | arg\_iter |
|  | |
| class | arg\_lbfgs |
|  | |
| class | arg\_max\_depth |
|  | |
| class | arg\_method |
|  | |
| class | arg\_metric |
|  | |
| class | arg\_newton |
|  | |
| class | arg\_num\_samples |
|  | |
| class | arg\_num\_warmup |
|  | |
| class | arg\_nuts |
|  | |
| class | arg\_optimize |
|  | |
| class | arg\_optimize\_algo |
|  | |
| class | arg\_output |
|  | |
| class | arg\_output\_file |
|  | |
| class | arg\_random |
|  | |
| class | arg\_refresh |
|  | |
| class | arg\_rwm |
|  | |
| class | arg\_sample |
|  | |
| class | arg\_sample\_algo |
|  | |
| class | arg\_save\_iterations |
|  | |
| class | arg\_save\_warmup |
|  | |
| class | arg\_seed |
|  | |
| class | arg\_static |
|  | |
| class | arg\_stepsize |
|  | |
| class | arg\_stepsize\_jitter |
|  | |
| class | arg\_test |
|  | |
| class | arg\_test\_grad\_eps |
|  | |
| class | arg\_test\_grad\_err |
|  | |
| class | arg\_test\_gradient |
|  | |
| class | arg\_thin |
|  | |
| class | arg\_tolerance |
|  | |
| class | arg\_unit\_e |
|  | |
| class | argument |
|  | |
| class | argument\_parser |
|  | |
| class | argument\_probe |
|  | |
| class | categorical\_argument |
|  | |
| class | list\_argument |
|  | |
| struct | type\_name |
|  | |
| struct | type\_name< int > |
|  | |
| struct | type\_name< unsigned int > |
|  | |
| struct | type\_name< double > |
|  | |
| struct | type\_name< bool > |
|  | |
| struct | type\_name< std::string > |
|  | |
| class | singleton\_argument |
|  | |
| class | unvalued\_argument |
|  | |
| class | valued\_argument |
|  | |
| struct | nil |
|  | Placeholder struct for boost::variant default ctors. More... |
|  | |
| struct | expr\_type |
|  | |
| class | function\_signatures |
|  | |
| struct | statements |
|  | |
| struct | distribution |
|  | |
| struct | expression\_type\_vis |
|  | |
| struct | expression |
|  | |
| struct | printable |
|  | |
| struct | is\_nil\_op |
|  | |
| struct | variable\_dims |
|  | |
| struct | int\_literal |
|  | |
| struct | double\_literal |
|  | |
| struct | array\_literal |
|  | |
| struct | variable |
|  | |
| struct | integrate\_ode |
|  | |
| struct | fun |
|  | |
| struct | index\_op |
|  | |
| struct | binary\_op |
|  | |
| struct | unary\_op |
|  | |
| struct | range |
|  | |
| struct | base\_var\_decl |
|  | |
| struct | variable\_map |
|  | |
| struct | int\_var\_decl |
|  | |
| struct | double\_var\_decl |
|  | |
| struct | unit\_vector\_var\_decl |
|  | |
| struct | simplex\_var\_decl |
|  | |
| struct | ordered\_var\_decl |
|  | |
| struct | positive\_ordered\_var\_decl |
|  | |
| struct | vector\_var\_decl |
|  | |
| struct | row\_vector\_var\_decl |
|  | |
| struct | matrix\_var\_decl |
|  | |
| struct | cholesky\_factor\_var\_decl |
|  | |
| struct | cholesky\_corr\_var\_decl |
|  | |
| struct | cov\_matrix\_var\_decl |
|  | |
| struct | corr\_matrix\_var\_decl |
|  | |
| struct | name\_vis |
|  | |
| struct | var\_decl |
|  | |
| struct | statement |
|  | |
| struct | is\_no\_op\_statement\_vis |
|  | |
| struct | returns\_type\_vis |
|  | |
| struct | increment\_log\_prob\_statement |
|  | |
| struct | for\_statement |
|  | |
| struct | conditional\_statement |
|  | |
| struct | while\_statement |
|  | |
| struct | print\_statement |
|  | |
| struct | reject\_statement |
|  | |
| struct | return\_statement |
|  | |
| struct | no\_op\_statement |
|  | |
| struct | arg\_decl |
|  | |
| struct | function\_decl\_def |
|  | |
| struct | function\_decl\_defs |
|  | |
| struct | program |
|  | |
| struct | sample |
|  | |
| struct | assignment |
|  | |
| struct | contains\_var |
|  | |
| struct | contains\_nonparam\_var |
|  | |
| struct | error\_codes |
|  | |
| struct | visgen |
|  | generic visitor with output for extension More... |
|  | |
| struct | expression\_visgen |
|  | |
| struct | printable\_visgen |
|  | |
| struct | var\_size\_validating\_visgen |
|  | |
| struct | var\_resizing\_visgen |
|  | |
| struct | init\_local\_var\_visgen |
|  | |
| struct | validate\_var\_decl\_visgen |
|  | |
| struct | member\_var\_decl\_visgen |
|  | |
| struct | local\_var\_decl\_visgen |
|  | |
| struct | generate\_local\_var\_init\_nan\_visgen |
|  | |
| struct | generate\_init\_vars\_visgen |
|  | |
| struct | validate\_transformed\_params\_visgen |
|  | |
| struct | statement\_visgen |
|  | |
| struct | dump\_member\_var\_visgen |
|  | |
| struct | set\_param\_ranges\_visgen |
|  | |
| struct | generate\_init\_visgen |
|  | |
| struct | write\_dims\_visgen |
|  | |
| struct | write\_param\_names\_visgen |
|  | |
| struct | write\_csv\_header\_visgen |
|  | |
| struct | constrained\_param\_names\_visgen |
|  | |
| struct | unconstrained\_param\_names\_visgen |
|  | |
| struct | write\_csv\_visgen |
|  | |
| struct | write\_csv\_vars\_visgen |
|  | |
| struct | write\_array\_visgen |
|  | |
| struct | write\_array\_vars\_visgen |
|  | |
| struct | bare\_type\_grammar |
|  | |
| struct | term\_grammar |
|  | |
| struct | expression\_grammar |
|  | |
| struct | expression07\_grammar |
|  | |
| struct | validate\_expr\_type3 |
|  | |
| struct | set\_fun\_type3 |
|  | |
| struct | addition\_expr3 |
|  | |
| struct | subtraction\_expr3 |
|  | |
| struct | set\_fun\_type2 |
|  | |
| struct | binary\_op\_expr |
|  | |
| struct | functions\_grammar |
|  | |
| struct | validate\_non\_void\_arg\_function |
|  | |
| struct | set\_void\_function |
|  | |
| struct | set\_allows\_sampling\_origin |
|  | |
| struct | validate\_declarations |
|  | |
| struct | add\_function\_signature |
|  | |
| struct | validate\_return\_type |
|  | |
| struct | scope\_lp |
|  | |
| struct | unscope\_variables |
|  | |
| struct | add\_fun\_var |
|  | |
| struct | program\_grammar |
|  | |
| struct | statement\_grammar |
|  | |
| struct | statement\_2\_grammar |
|  | |
| struct | add\_conditional\_condition |
|  | |
| struct | add\_conditional\_body |
|  | |
| struct | validate\_return\_allowed |
|  | |
| struct | validate\_void\_return\_allowed |
|  | |
| struct | validate\_assignment |
|  | |
| struct | validate\_sample |
|  | |
| struct | expression\_as\_statement |
|  | |
| struct | unscope\_locals |
|  | |
| struct | add\_while\_condition |
|  | |
| struct | add\_while\_body |
|  | |
| struct | add\_loop\_identifier |
|  | |
| struct | remove\_loop\_identifier |
|  | |
| struct | validate\_int\_expr2 |
|  | |
| struct | validate\_allow\_sample |
|  | |
| struct | validate\_non\_void\_expression |
|  | |
| struct | validate\_integrate\_ode |
|  | |
| struct | set\_fun\_type |
|  | |
| struct | set\_fun\_type\_named |
|  | |
| struct | exponentiation\_expr |
|  | |
| struct | multiplication\_expr |
|  | |
| struct | division\_expr |
|  | |
| struct | modulus\_expr |
|  | |
| struct | left\_division\_expr |
|  | |
| struct | elt\_multiplication\_expr |
|  | |
| struct | elt\_division\_expr |
|  | |
| struct | negate\_expr |
|  | |
| struct | logical\_negate\_expr |
|  | |
| struct | transpose\_expr |
|  | |
| struct | add\_expression\_dimss |
|  | |
| struct | set\_var\_type |
|  | |
| struct | validate\_int\_expr3 |
|  | |
| struct | var\_decls\_grammar |
|  | |
| struct | whitespace\_grammar |
|  | |

|  |  |
| --- | --- |
| Typedefs | |
| typedef singleton\_argument  < double > | real\_argument |
|  | |
| typedef singleton\_argument< int > | int\_argument |
|  | |
| typedef singleton\_argument  < unsigned int > | u\_int\_argument |
|  | |
| typedef singleton\_argument< bool > | bool\_argument |
|  | |
| typedef singleton\_argument  < std::string > | string\_argument |
|  | |
| typedef int | base\_expr\_type |
|  | |
| typedef std::pair< expr\_type,   std::vector< expr\_type > > | function\_signature\_t |
|  | |
| typedef int | var\_origin |
|  | |
| typedef std::string::const\_iterator | input\_iterator\_t |
|  | |
| typedef   boost::spirit::line\_pos\_iterator  < input\_iterator\_t > | pos\_iterator\_t |
|  | |

|  |  |
| --- | --- |
| Functions | |
| std::ostream & | write\_base\_expr\_type (std::ostream &o, base\_expr\_type type) |
|  | |
| std::ostream & | operator<< (std::ostream &o, const expr\_type &et) |
|  | |
| expr\_type | promote\_primitive (const expr\_type &et) |
|  | |
| expr\_type | promote\_primitive (const expr\_type &et1, const expr\_type &et2) |
|  | |
| bool | is\_nil (const expression &e) |
|  | |
| size\_t | total\_dims (const std::vector< std::vector< expression > > &dimss) |
|  | |
| expr\_type | infer\_type\_indexing (const base\_expr\_type &expr\_base\_type, size\_t num\_expr\_dims, size\_t num\_index\_dims) |
|  | |
| expr\_type | infer\_type\_indexing (const expression &expr, size\_t num\_index\_dims) |
|  | |
| void | print\_var\_origin (std::ostream &o, const var\_origin &vo) |
|  | |
| bool | returns\_type (const expr\_type &return\_type, const statement &statement, std::ostream &error\_msgs) |
|  | |
| void | generate\_expression (const expression &e, std::ostream &o) |
|  | |
| bool | has\_rng\_suffix (const std::string &s) |
|  | |
| bool | has\_lp\_suffix (const std::string &s) |
|  | |
| bool | is\_user\_defined (const std::string &name, const std::vector< expression > &args) |
|  | |
| bool | is\_user\_defined\_prob\_function (const std::string &name, const expression &variate, const std::vector< expression > &params) |
|  | |
| bool | is\_user\_defined (const fun &fx) |
|  | |
| bool | has\_var (const expression &e, const variable\_map &var\_map) |
|  | |
| bool | has\_non\_param\_var (const expression &e, const variable\_map &var\_map) |
|  | |
| bool | is\_assignable (const expr\_type &l\_type, const expr\_type &r\_type, const std::string &failure\_message, std::ostream &error\_msgs) |
|  | |
| bool | ends\_with (const std::string &suffix, const std::string &s) |
|  | |
| bool | is\_linear\_function (const std::string &name) |
|  | |
| bool | compile (std::ostream \*msgs, std::istream &stan\_gm\_in, std::ostream &cpp\_out, const std::string &model\_name, const std::string &in\_file\_name="input") |
|  | Read a Stan model specification from the specified input, parse it, and write the C++ code for it to the specified output. More... |
|  | |
| const std::string | EOL ("\n") |
|  | |
| const std::string | EOL2 ("\n\n") |
|  | |
| const std::string | INDENT (" ") |
|  | |
| const std::string | INDENT2 (" ") |
|  | |
| const std::string | INDENT3 (" ") |
|  | |
| template<typename D > | |
| bool | has\_lub (const D &x) |
|  | |
| template<typename D > | |
| bool | has\_ub (const D &x) |
|  | |
| template<typename D > | |
| bool | has\_lb (const D &x) |
|  | |
| template<typename T > | |
| std::string | to\_string (T i) |
|  | |
| void | generate\_indent (size\_t indent, std::ostream &o) |
|  | |
| void | generate\_void\_statement (const std::string &name, const size\_t indent, std::ostream &o) |
|  | |
| void | generate\_start\_namespace (std::string name, std::ostream &o) |
|  | |
| void | generate\_end\_namespace (std::ostream &o) |
|  | |
| void | generate\_comment (std::string const &msg, int indent, std::ostream &o) |
|  | |
| template<bool isLHS> | |
| void | generate\_indexed\_expr (const std::string &expr, const std::vector< expression > indexes, base\_expr\_type base\_type, size\_t e\_num\_dims, std::ostream &o) |
|  | |
| void | generate\_type (const std::string &base\_type, const std::vector< expression > &, size\_t end, std::ostream &o) |
|  | |
| static void | print\_string\_literal (std::ostream &o, const std::string &s) |
|  | |
| static void | print\_quoted\_expression (std::ostream &o, const expression &e) |
|  | |
| void | generate\_printable (const printable &p, std::ostream &o) |
|  | |
| void | generate\_using (const std::string &type, std::ostream &o) |
|  | |
| void | generate\_using\_namespace (const std::string &ns, std::ostream &o) |
|  | |
| void | generate\_usings (std::ostream &o) |
|  | |
| void | generate\_typedef (const std::string &type, const std::string &abbrev, std::ostream &o) |
|  | |
| void | generate\_typedefs (std::ostream &o) |
|  | |
| void | generate\_include (const std::string &lib\_name, std::ostream &o) |
|  | |
| void | generate\_includes (std::ostream &o) |
|  | |
| void | generate\_version\_comment (std::ostream &o) |
|  | |
| void | generate\_class\_decl (const std::string &model\_name, std::ostream &o) |
|  | |
| void | generate\_end\_class\_decl (std::ostream &o) |
|  | |
| void | generate\_initializer (std::ostream &o, const std::string &base\_type, const std::vector< expression > &dims, const expression &type\_arg1=expression(), const expression &type\_arg2=expression()) |
|  | |
| void | generate\_validate\_context\_size (std::ostream &o, const std::string &stage, const std::string &var\_name, const std::string &base\_type, const std::vector< expression > &dims, const expression &type\_arg1=expression(), const expression &type\_arg2=expression()) |
|  | |
| void | generate\_validate\_positive (const std::string &var\_name, const expression &expr, std::ostream &o) |
|  | |
| void | generate\_initialization (std::ostream &o, const std::string &var\_name, const std::string &base\_type, const std::vector< expression > &dims, const expression &type\_arg1=expression(), const expression &type\_arg2=expression()) |
|  | |
| void | generate\_var\_resizing (const std::vector< var\_decl > &vs, std::ostream &o) |
|  | |
| const std::vector< expression > | EMPTY\_EXP\_VECTOR (0) |
|  | |
| void | generate\_local\_var\_inits (std::vector< var\_decl > vs, bool is\_var, bool declare\_vars, std::ostream &o) |
|  | |
| void | generate\_public\_decl (std::ostream &o) |
|  | |
| void | generate\_private\_decl (std::ostream &o) |
|  | |
| void | generate\_validate\_var\_decl (const var\_decl &decl, int indent, std::ostream &o) |
|  | |
| void | generate\_validate\_var\_decls (const std::vector< var\_decl > decls, int indent, std::ostream &o) |
|  | |
| void | generate\_member\_var\_decls (const std::vector< var\_decl > &vs, int indent, std::ostream &o) |
|  | |
| void | generate\_local\_var\_decls (const std::vector< var\_decl > &vs, int indent, std::ostream &o, bool is\_var, bool is\_fun\_return) |
|  | |
| void | generate\_local\_var\_init\_nan (const std::vector< var\_decl > &vs, int indent, std::ostream &o, bool is\_var, bool is\_fun\_return) |
|  | |
| void | generate\_init\_vars (const std::vector< var\_decl > &vs, int indent, std::ostream &o) |
|  | |
| void | generate\_validate\_transformed\_params (const std::vector< var\_decl > &vs, int indent, std::ostream &o) |
|  | |
| void | generate\_statement (statement const &s, int indent, std::ostream &o, bool include\_sampling, bool is\_var, bool is\_fun\_return) |
|  | |
| void | generate\_statements (const std::vector< statement > &ss, int indent, std::ostream &o, bool include\_sampling, bool is\_var, bool is\_fun\_return) |
|  | |
| void | generate\_log\_prob (program const &p, std::ostream &o) |
|  | |
| void | suppress\_warning (const std::string &indent, const std::string &var\_name, std::ostream &o) |
|  | |
| void | generate\_member\_var\_inits (const std::vector< var\_decl > &vs, std::ostream &o) |
|  | |
| void | generate\_destructor (const std::string &model\_name, std::ostream &o) |
|  | |
| void | generate\_set\_param\_ranges (const std::vector< var\_decl > &var\_decls, std::ostream &o) |
|  | |
| void | generate\_constructor (const program &prog, const std::string &model\_name, std::ostream &o) |
|  | |
| void | generate\_init\_method (const std::vector< var\_decl > &vs, std::ostream &o) |
|  | |
| void | generate\_dims\_method (const program &prog, std::ostream &o) |
|  | |
| void | generate\_param\_names\_method (const program &prog, std::ostream &o) |
|  | |
| void | generate\_write\_csv\_header\_method (const program &prog, std::ostream &o) |
|  | |
| void | generate\_constrained\_param\_names\_method (const program &prog, std::ostream &o) |
|  | |
| void | generate\_unconstrained\_param\_names\_method (const program &prog, std::ostream &o) |
|  | |
| void | generate\_write\_csv\_method (const program &prog, const std::string &model\_name, std::ostream &o) |
|  | |
| void | generate\_write\_array\_method (const program &prog, const std::string &model\_name, std::ostream &o) |
|  | |
| void | generate\_model\_name\_method (const std::string &model\_name, std::ostream &out) |
|  | |
| void | generate\_model\_typedef (const std::string &model\_name, std::ostream &out) |
|  | |
| void | generate\_bare\_type (const expr\_type &t, const std::string &scalar\_t\_name, std::ostream &out) |
|  | |
| void | generate\_arg\_decl (bool gen\_const, bool gen\_ref, const arg\_decl &decl, const std::string &scalar\_t\_name, std::ostream &out) |
|  | |
| bool | has\_only\_int\_args (const function\_decl\_def &fun) |
|  | |
| std::string | fun\_scalar\_type (const function\_decl\_def &fun, bool is\_lp) |
|  | |
| bool | needs\_template\_params (const function\_decl\_def &fun) |
|  | |
| void | generate\_function\_template\_parameters (const function\_decl\_def &fun, bool is\_rng, bool is\_lp, bool is\_log, std::ostream &out) |
|  | |
| void | generate\_function\_inline\_return\_type (const function\_decl\_def &fun, const std::string &scalar\_t\_name, std::ostream &out) |
|  | |
| void | generate\_function\_name (const function\_decl\_def &fun, std::ostream &out) |
|  | |
| void | generate\_function\_arguments (const function\_decl\_def &fun, bool is\_rng, bool is\_lp, bool is\_log, std::ostream &out) |
|  | |
| void | generate\_functor\_arguments (const function\_decl\_def &fun, bool is\_rng, bool is\_lp, bool is\_log, std::ostream &out) |
|  | |
| void | generate\_function\_body (const function\_decl\_def &fun, const std::string &scalar\_t\_name, std::ostream &out) |
|  | |
| void | generate\_propto\_default\_function\_body (const function\_decl\_def &fun, std::ostream &out) |
|  | |
| void | generate\_propto\_default\_function (const function\_decl\_def &fun, const std::string &scalar\_t\_name, std::ostream &out) |
|  | |
| void | generate\_function (const function\_decl\_def &fun, std::ostream &out) |
|  | Generate the specified function and optionally its default for propto=false for functions ending in \_log. More... |
|  | |
| void | generate\_function\_functor (const function\_decl\_def &fun, std::ostream &out) |
|  | |
| void | generate\_functions (const std::vector< function\_decl\_def > &funs, std::ostream &out) |
|  | |
| void | generate\_cpp (const program &prog, const std::string &model\_name, std::ostream &out) |
|  | |
| bool | is\_space (char c) |
|  | |
| bool | is\_nonempty (std::string &s) |
|  | |
| bool | parse (std::ostream \*output\_stream, std::istream &input, const std::string &filename, const std::string &model\_name, program &result) |
|  | |

|  |  |
| --- | --- |
| Variables | |
| const int | VOID\_T = 0 |
|  | |
| const int | INT\_T = 1 |
|  | |
| const int | DOUBLE\_T = 2 |
|  | |
| const int | VECTOR\_T = 3 |
|  | |
| const int | ROW\_VECTOR\_T = 4 |
|  | |
| const int | MATRIX\_T = 5 |
|  | |
| const int | ILL\_FORMED\_T = 6 |
|  | |
| const int | model\_name\_origin = 0 |
|  | |
| const int | data\_origin = 1 |
|  | |
| const int | transformed\_data\_origin = 2 |
|  | |
| const int | parameter\_origin = 3 |
|  | |
| const int | transformed\_parameter\_origin = 4 |
|  | |
| const int | derived\_origin = 5 |
|  | |
| const int | local\_origin = 6 |
|  | |
| const int | function\_argument\_origin = 7 |
|  | |
| const int | function\_argument\_origin\_lp = 8 |
|  | |
| const int | function\_argument\_origin\_rng = 9 |
|  | |
| const int | void\_function\_argument\_origin = 10 |
|  | |
| const int | void\_function\_argument\_origin\_lp = 11 |
|  | |
| const int | void\_function\_argument\_origin\_rng = 12 |
|  | |
| boost::phoenix::function  < validate\_expr\_type3 > | validate\_expr\_type3\_f |
|  | |
| boost::phoenix::function  < set\_fun\_type3 > | set\_fun\_type3\_f |
|  | |
| boost::phoenix::function  < addition\_expr3 > | addition3\_f |
|  | |
| boost::phoenix::function  < subtraction\_expr3 > | subtraction3\_f |
|  | |
| boost::phoenix::function  < set\_fun\_type2 > | set\_fun\_type2\_f |
|  | |
| boost::phoenix::function  < binary\_op\_expr > | binary\_op\_f |
|  | |
| boost::phoenix::function  < validate\_non\_void\_arg\_function > | validate\_non\_void\_arg\_f |
|  | |
| boost::phoenix::function  < set\_void\_function > | set\_void\_function\_f |
|  | |
| boost::phoenix::function  < set\_allows\_sampling\_origin > | set\_allows\_sampling\_origin\_f |
|  | |
| boost::phoenix::function  < validate\_declarations > | validate\_declarations\_f |
|  | |
| boost::phoenix::function  < add\_function\_signature > | add\_function\_signature\_f |
|  | |
| boost::phoenix::function  < validate\_return\_type > | validate\_return\_type\_f |
|  | |
| boost::phoenix::function  < scope\_lp > | scope\_lp\_f |
|  | |
| boost::phoenix::function  < unscope\_variables > | unscope\_variables\_f |
|  | |
| boost::phoenix::function  < add\_fun\_var > | add\_fun\_var\_f |
|  | |
| boost::phoenix::function  < add\_conditional\_condition > | add\_conditional\_condition\_f |
|  | |
| boost::phoenix::function  < add\_conditional\_body > | add\_conditional\_body\_f |
|  | |
| boost::phoenix::function  < validate\_return\_allowed > | validate\_return\_allowed\_f |
|  | |
| boost::phoenix::function  < validate\_void\_return\_allowed > | validate\_void\_return\_allowed\_f |
|  | |
| boost::phoenix::function  < validate\_assignment > | validate\_assignment\_f |
|  | |
| boost::phoenix::function  < validate\_sample > | validate\_sample\_f |
|  | |
| boost::phoenix::function  < expression\_as\_statement > | expression\_as\_statement\_f |
|  | |
| boost::phoenix::function  < unscope\_locals > | unscope\_locals\_f |
|  | |
| boost::phoenix::function  < add\_while\_condition > | add\_while\_condition\_f |
|  | |
| boost::phoenix::function  < add\_while\_body > | add\_while\_body\_f |
|  | |
| boost::phoenix::function  < add\_loop\_identifier > | add\_loop\_identifier\_f |
|  | |
| boost::phoenix::function  < remove\_loop\_identifier > | remove\_loop\_identifier\_f |
|  | |
| boost::phoenix::function  < validate\_int\_expr2 > | validate\_int\_expr2\_f |
|  | |
| boost::phoenix::function  < validate\_allow\_sample > | validate\_allow\_sample\_f |
|  | |
| boost::phoenix::function  < validate\_non\_void\_expression > | validate\_non\_void\_expression\_f |
|  | |
| boost::phoenix::function  < validate\_integrate\_ode > | validate\_integrate\_ode\_f |
|  | |
| boost::phoenix::function  < set\_fun\_type > | set\_fun\_type\_f |
|  | |
| boost::phoenix::function  < set\_fun\_type\_named > | set\_fun\_type\_named\_f |
|  | |
| boost::phoenix::function  < exponentiation\_expr > | exponentiation\_f |
|  | |
| boost::phoenix::function  < multiplication\_expr > | multiplication\_f |
|  | |
| boost::phoenix::function  < division\_expr > | division\_f |
|  | |
| boost::phoenix::function  < modulus\_expr > | modulus\_f |
|  | |
| boost::phoenix::function  < left\_division\_expr > | left\_division\_f |
|  | |
| boost::phoenix::function  < elt\_multiplication\_expr > | elt\_multiplication\_f |
|  | |
| boost::phoenix::function  < elt\_division\_expr > | elt\_division\_f |
|  | |
| boost::phoenix::function  < negate\_expr > | negate\_expr\_f |
|  | |
| boost::phoenix::function  < logical\_negate\_expr > | logical\_negate\_expr\_f |
|  | |
| boost::phoenix::function  < transpose\_expr > | transpose\_f |
|  | |
| boost::phoenix::function  < add\_expression\_dimss > | add\_expression\_dimss\_f |
|  | |
| boost::phoenix::function  < set\_var\_type > | set\_var\_type\_f |
|  | |
| boost::phoenix::function  < validate\_int\_expr3 > | validate\_int\_expr3\_f |
|  | |

## Detailed Description

Directed graphical model compiler.

## Typedef Documentation

typedef int stan::gm::base\_expr\_type

Definition at line 60 of file ast.hpp.

typedef singleton\_argument<bool> stan::gm::bool\_argument

Definition at line 167 of file singleton\_argument.hpp.

typedef std::pair<expr\_type, std::vector<expr\_type> > stan::gm::function\_signature\_t

Definition at line 104 of file ast.hpp.

typedef std::string::const\_iterator stan::gm::input\_iterator\_t

Definition at line 9 of file iterator\_typedefs.hpp.

typedef singleton\_argument<int> stan::gm::int\_argument

Definition at line 165 of file singleton\_argument.hpp.

typedef boost::spirit::line\_pos\_iterator<input\_iterator\_t> stan::gm::pos\_iterator\_t

Definition at line 10 of file iterator\_typedefs.hpp.

typedef singleton\_argument<double> stan::gm::real\_argument

Definition at line 164 of file singleton\_argument.hpp.

typedef singleton\_argument<std::string> stan::gm::string\_argument

Definition at line 168 of file singleton\_argument.hpp.

typedef singleton\_argument<unsigned int> stan::gm::u\_int\_argument

Definition at line 166 of file singleton\_argument.hpp.

typedef int stan::gm::var\_origin

Definition at line 411 of file ast.hpp.

## Function Documentation

|  |  |  |  |
| --- | --- | --- | --- |
| bool stan::gm::compile | ( | std::ostream \* | *msgs*, |
|  |  | std::istream & | *stan\_gm\_in*, |
|  |  | std::ostream & | *cpp\_out*, |
|  |  | const std::string & | *model\_name*, |
|  |  | const std::string & | *in\_file\_name* = `"input"` |
|  | ) |  |  |

Read a Stan model specification from the specified input, parse it, and write the C++ code for it to the specified output.

Parameters
:   |  |  |
    | --- | --- |
    | msgs | Output stream for warning messages |
    | stan\_gm\_in | Stan model specification |
    | cpp\_out | C++ code output stream |
    | model\_name | Name of model class |
    | include\_main | Indicates whether to generate a main function |
    | in\_file\_name | Name of input file to use in error messages; defaults to `input`. |

Returns
:   `false` if code could not be generated due to syntax error in the Stan model; `true` otherwise.

Parameters
:   |  |  |
    | --- | --- |
    | msgs |  |
    | stan\_gm\_in |  |
    | cpp\_out |  |
    | model\_name |  |
    | in\_file\_name |  |

Definition at line 37 of file compiler.hpp.

|  |  |  |  |  |  |
| --- | --- | --- | --- | --- | --- |
| const std::vector<expression> stan::gm::EMPTY\_EXP\_VECTOR | ( | 0 |  | ) |  |

|  |  |  |  |
| --- | --- | --- | --- |
| bool stan::gm::ends\_with | ( | const std::string & | *suffix*, |
|  |  | const std::string & | *s* |
|  | ) |  |  |

Definition at line 1477 of file ast\_def.cpp.

|  |  |  |  |  |  |
| --- | --- | --- | --- | --- | --- |
| const std::string stan::gm::EOL | ( | "\n" |  | ) |  |

|  |  |  |  |  |  |
| --- | --- | --- | --- | --- | --- |
| const std::string stan::gm::EOL2 | ( | "\n\n" |  | ) |  |

|  |  |  |  |
| --- | --- | --- | --- |
| std::string stan::gm::fun\_scalar\_type | ( | const function\_decl\_def & | *fun*, |
|  |  | bool | *is\_lp* |
|  | ) |  |  |

Definition at line 4309 of file generator.hpp.

|  |  |  |  |
| --- | --- | --- | --- |
| void stan::gm::generate\_arg\_decl | ( | bool | *gen\_const*, |
|  |  | bool | *gen\_ref*, |
|  |  | const arg\_decl & | *decl*, |
|  |  | const std::string & | *scalar\_t\_name*, |
|  |  | std::ostream & | *out* |
|  | ) |  |  |

Definition at line 4289 of file generator.hpp.

|  |  |  |  |
| --- | --- | --- | --- |
| void stan::gm::generate\_bare\_type | ( | const expr\_type & | *t*, |
|  |  | const std::string & | *scalar\_t\_name*, |
|  |  | std::ostream & | *out* |
|  | ) |  |  |

Definition at line 4241 of file generator.hpp.

|  |  |  |  |
| --- | --- | --- | --- |
| void stan::gm::generate\_class\_decl | ( | const std::string & | *model\_name*, |
|  |  | std::ostream & | *o* |
|  | ) |  |  |

Definition at line 339 of file generator.hpp.

|  |  |  |  |
| --- | --- | --- | --- |
| void stan::gm::generate\_comment | ( | std::string const & | *msg*, |
|  |  | int | *indent*, |
|  |  | std::ostream & | *o* |
|  | ) |  |  |

Definition at line 79 of file generator.hpp.

|  |  |  |  |
| --- | --- | --- | --- |
| void stan::gm::generate\_constrained\_param\_names\_method | ( | const program & | *prog*, |
|  |  | std::ostream & | *o* |
|  | ) |  |  |

Definition at line 3308 of file generator.hpp.

|  |  |  |  |
| --- | --- | --- | --- |
| void stan::gm::generate\_constructor | ( | const program & | *prog*, |
|  |  | const std::string & | *model\_name*, |
|  |  | std::ostream & | *o* |
|  | ) |  |  |

Definition at line 2517 of file generator.hpp.

|  |  |  |  |
| --- | --- | --- | --- |
| void stan::gm::generate\_cpp | ( | const program & | *prog*, |
|  |  | const std::string & | *model\_name*, |
|  |  | std::ostream & | *out* |
|  | ) |  |  |

Definition at line 4614 of file generator.hpp.

|  |  |  |  |
| --- | --- | --- | --- |
| void stan::gm::generate\_destructor | ( | const std::string & | *model\_name*, |
|  |  | std::ostream & | *o* |
|  | ) |  |  |

Definition at line 2320 of file generator.hpp.

|  |  |  |  |
| --- | --- | --- | --- |
| void stan::gm::generate\_dims\_method | ( | const program & | *prog*, |
|  |  | std::ostream & | *o* |
|  | ) |  |  |

Definition at line 2920 of file generator.hpp.

|  |  |  |  |  |  |
| --- | --- | --- | --- | --- | --- |
| void stan::gm::generate\_end\_class\_decl | ( | std::ostream & | *o* | ) |  |

Definition at line 344 of file generator.hpp.

|  |  |  |  |  |  |
| --- | --- | --- | --- | --- | --- |
| void stan::gm::generate\_end\_namespace | ( | std::ostream & | *o* | ) |  |

Definition at line 75 of file generator.hpp.

|  |  |  |  |
| --- | --- | --- | --- |
| void stan::gm::generate\_expression | ( | const expression & | *e*, |
|  |  | std::ostream & | *o* |
|  | ) |  |  |

Definition at line 249 of file generator.hpp.

|  |  |  |  |
| --- | --- | --- | --- |
| void stan::gm::generate\_function | ( | const function\_decl\_def & | *fun*, |
|  |  | std::ostream & | *out* |
|  | ) |  |  |

Generate the specified function and optionally its default for propto=false for functions ending in \_log.

Exact behavior differs for unmarked functions, and functions ending in one of "\_rng", "\_lp", or "\_log".

Parameters
:   |  |  |  |
    | --- | --- | --- |
    | [in] | fun | function AST object |
    | [in,out] | out | output stream to which function definition is written |

Definition at line 4543 of file generator.hpp.

|  |  |  |  |
| --- | --- | --- | --- |
| void stan::gm::generate\_function\_arguments | ( | const function\_decl\_def & | *fun*, |
|  |  | bool | *is\_rng*, |
|  |  | bool | *is\_lp*, |
|  |  | bool | *is\_log*, |
|  |  | std::ostream & | *out* |
|  | ) |  |  |

Definition at line 4418 of file generator.hpp.

|  |  |  |  |
| --- | --- | --- | --- |
| void stan::gm::generate\_function\_body | ( | const function\_decl\_def & | *fun*, |
|  |  | const std::string & | *scalar\_t\_name*, |
|  |  | std::ostream & | *out* |
|  | ) |  |  |

Definition at line 4473 of file generator.hpp.

|  |  |  |  |
| --- | --- | --- | --- |
| void stan::gm::generate\_function\_functor | ( | const function\_decl\_def & | *fun*, |
|  |  | std::ostream & | *out* |
|  | ) |  |  |

Definition at line 4564 of file generator.hpp.

|  |  |  |  |
| --- | --- | --- | --- |
| void stan::gm::generate\_function\_inline\_return\_type | ( | const function\_decl\_def & | *fun*, |
|  |  | const std::string & | *scalar\_t\_name*, |
|  |  | std::ostream & | *out* |
|  | ) |  |  |

Definition at line 4404 of file generator.hpp.

|  |  |  |  |
| --- | --- | --- | --- |
| void stan::gm::generate\_function\_name | ( | const function\_decl\_def & | *fun*, |
|  |  | std::ostream & | *out* |
|  | ) |  |  |

Definition at line 4412 of file generator.hpp.

|  |  |  |  |
| --- | --- | --- | --- |
| void stan::gm::generate\_function\_template\_parameters | ( | const function\_decl\_def & | *fun*, |
|  |  | bool | *is\_rng*, |
|  |  | bool | *is\_lp*, |
|  |  | bool | *is\_log*, |
|  |  | std::ostream & | *out* |
|  | ) |  |  |

Definition at line 4356 of file generator.hpp.

|  |  |  |  |
| --- | --- | --- | --- |
| void stan::gm::generate\_functions | ( | const std::vector< function\_decl\_def > & | *funs*, |
|  |  | std::ostream & | *out* |
|  | ) |  |  |

Definition at line 4606 of file generator.hpp.

|  |  |  |  |
| --- | --- | --- | --- |
| void stan::gm::generate\_functor\_arguments | ( | const function\_decl\_def & | *fun*, |
|  |  | bool | *is\_rng*, |
|  |  | bool | *is\_lp*, |
|  |  | bool | *is\_log*, |
|  |  | std::ostream & | *out* |
|  | ) |  |  |

Definition at line 4447 of file generator.hpp.

|  |  |  |  |
| --- | --- | --- | --- |
| void stan::gm::generate\_include | ( | const std::string & | *lib\_name*, |
|  |  | std::ostream & | *o* |
|  | ) |  |  |

Definition at line 323 of file generator.hpp.

|  |  |  |  |  |  |
| --- | --- | --- | --- | --- | --- |
| void stan::gm::generate\_includes | ( | std::ostream & | *o* | ) |  |

Definition at line 327 of file generator.hpp.

|  |  |  |  |
| --- | --- | --- | --- |
| void stan::gm::generate\_indent | ( | size\_t | *indent*, |
|  |  | std::ostream & | *o* |
|  | ) |  |  |

Definition at line 50 of file generator.hpp.

template<bool isLHS>

|  |  |  |  |
| --- | --- | --- | --- |
| void stan::gm::generate\_indexed\_expr | ( | const std::string & | *expr*, |
|  |  | const std::vector< expression > | *indexes*, |
|  |  | base\_expr\_type | *base\_type*, |
|  |  | size\_t | *e\_num\_dims*, |
|  |  | std::ostream & | *o* |
|  | ) |  |  |

Definition at line 87 of file generator.hpp.

|  |  |  |  |
| --- | --- | --- | --- |
| void stan::gm::generate\_init\_method | ( | const std::vector< var\_decl > & | *vs*, |
|  |  | std::ostream & | *o* |
|  | ) |  |  |

Definition at line 2795 of file generator.hpp.

|  |  |  |  |
| --- | --- | --- | --- |
| void stan::gm::generate\_init\_vars | ( | const std::vector< var\_decl > & | *vs*, |
|  |  | int | *indent*, |
|  |  | std::ostream & | *o* |
|  | ) |  |  |

Definition at line 1369 of file generator.hpp.

|  |  |  |  |
| --- | --- | --- | --- |
| void stan::gm::generate\_initialization | ( | std::ostream & | *o*, |
|  |  | const std::string & | *var\_name*, |
|  |  | const std::string & | *base\_type*, |
|  |  | const std::vector< expression > & | *dims*, |
|  |  | const expression & | *type\_arg1* = `expression()`, |
|  |  | const expression & | *type\_arg2* = `expression()` |
|  | ) |  |  |

Definition at line 469 of file generator.hpp.

|  |  |  |  |
| --- | --- | --- | --- |
| void stan::gm::generate\_initializer | ( | std::ostream & | *o*, |
|  |  | const std::string & | *base\_type*, |
|  |  | const std::vector< expression > & | *dims*, |
|  |  | const expression & | *type\_arg1* = `expression()`, |
|  |  | const expression & | *type\_arg2* = `expression()` |
|  | ) |  |  |

Definition at line 348 of file generator.hpp.

|  |  |  |  |
| --- | --- | --- | --- |
| void stan::gm::generate\_local\_var\_decls | ( | const std::vector< var\_decl > & | *vs*, |
|  |  | int | *indent*, |
|  |  | std::ostream & | *o*, |
|  |  | bool | *is\_var*, |
|  |  | bool | *is\_fun\_return* |
|  | ) |  |  |

Definition at line 1215 of file generator.hpp.

|  |  |  |  |
| --- | --- | --- | --- |
| void stan::gm::generate\_local\_var\_init\_nan | ( | const std::vector< var\_decl > & | *vs*, |
|  |  | int | *indent*, |
|  |  | std::ostream & | *o*, |
|  |  | bool | *is\_var*, |
|  |  | bool | *is\_fun\_return* |
|  | ) |  |  |

Definition at line 1295 of file generator.hpp.

|  |  |  |  |
| --- | --- | --- | --- |
| void stan::gm::generate\_local\_var\_inits | ( | std::vector< var\_decl > | *vs*, |
|  |  | bool | *is\_var*, |
|  |  | bool | *declare\_vars*, |
|  |  | std::ostream & | *o* |
|  | ) |  |  |

Definition at line 758 of file generator.hpp.

|  |  |  |  |
| --- | --- | --- | --- |
| void stan::gm::generate\_log\_prob | ( | program const & | *p*, |
|  |  | std::ostream & | *o* |
|  | ) |  |  |

Definition at line 1768 of file generator.hpp.

|  |  |  |  |
| --- | --- | --- | --- |
| void stan::gm::generate\_member\_var\_decls | ( | const std::vector< var\_decl > & | *vs*, |
|  |  | int | *indent*, |
|  |  | std::ostream & | *o* |
|  | ) |  |  |

Definition at line 987 of file generator.hpp.

|  |  |  |  |
| --- | --- | --- | --- |
| void stan::gm::generate\_member\_var\_inits | ( | const std::vector< var\_decl > & | *vs*, |
|  |  | std::ostream & | *o* |
|  | ) |  |  |

Definition at line 2313 of file generator.hpp.

|  |  |  |  |
| --- | --- | --- | --- |
| void stan::gm::generate\_model\_name\_method | ( | const std::string & | *model\_name*, |
|  |  | std::ostream & | *out* |
|  | ) |  |  |

Definition at line 4228 of file generator.hpp.

|  |  |  |  |
| --- | --- | --- | --- |
| void stan::gm::generate\_model\_typedef | ( | const std::string & | *model\_name*, |
|  |  | std::ostream & | *out* |
|  | ) |  |  |

Definition at line 4235 of file generator.hpp.

|  |  |  |  |
| --- | --- | --- | --- |
| void stan::gm::generate\_param\_names\_method | ( | const program & | *prog*, |
|  |  | std::ostream & | *o* |
|  | ) |  |  |

Definition at line 3001 of file generator.hpp.

|  |  |  |  |
| --- | --- | --- | --- |
| void stan::gm::generate\_printable | ( | const printable & | *p*, |
|  |  | std::ostream & | *o* |
|  | ) |  |  |

Definition at line 283 of file generator.hpp.

|  |  |  |  |  |  |
| --- | --- | --- | --- | --- | --- |
| void stan::gm::generate\_private\_decl | ( | std::ostream & | *o* | ) |  |

Definition at line 778 of file generator.hpp.

|  |  |  |  |
| --- | --- | --- | --- |
| void stan::gm::generate\_propto\_default\_function | ( | const function\_decl\_def & | *fun*, |
|  |  | const std::string & | *scalar\_t\_name*, |
|  |  | std::ostream & | *out* |
|  | ) |  |  |

Definition at line 4522 of file generator.hpp.

|  |  |  |  |
| --- | --- | --- | --- |
| void stan::gm::generate\_propto\_default\_function\_body | ( | const function\_decl\_def & | *fun*, |
|  |  | std::ostream & | *out* |
|  | ) |  |  |

Definition at line 4505 of file generator.hpp.

|  |  |  |  |  |  |
| --- | --- | --- | --- | --- | --- |
| void stan::gm::generate\_public\_decl | ( | std::ostream & | *o* | ) |  |

Definition at line 774 of file generator.hpp.

|  |  |  |  |
| --- | --- | --- | --- |
| void stan::gm::generate\_set\_param\_ranges | ( | const std::vector< var\_decl > & | *var\_decls*, |
|  |  | std::ostream & | *o* |
|  | ) |  |  |

Definition at line 2505 of file generator.hpp.

|  |  |  |  |
| --- | --- | --- | --- |
| void stan::gm::generate\_start\_namespace | ( | std::string | *name*, |
|  |  | std::ostream & | *o* |
|  | ) |  |  |

Definition at line 70 of file generator.hpp.

|  |  |  |  |
| --- | --- | --- | --- |
| void stan::gm::generate\_statement | ( | statement const & | *s*, |
|  |  | int | *indent*, |
|  |  | std::ostream & | *o*, |
|  |  | bool | *include\_sampling*, |
|  |  | bool | *is\_var*, |
|  |  | bool | *is\_fun\_return* |
|  | ) |  |  |

Definition at line 1746 of file generator.hpp.

|  |  |  |  |
| --- | --- | --- | --- |
| void stan::gm::generate\_statements | ( | const std::vector< statement > & | *ss*, |
|  |  | int | *indent*, |
|  |  | std::ostream & | *o*, |
|  |  | bool | *include\_sampling*, |
|  |  | bool | *is\_var*, |
|  |  | bool | *is\_fun\_return* |
|  | ) |  |  |

Definition at line 1756 of file generator.hpp.

|  |  |  |  |
| --- | --- | --- | --- |
| void stan::gm::generate\_type | ( | const std::string & | *base\_type*, |
|  |  | const std::vector< expression > & | , |
|  |  | size\_t | *end*, |
|  |  | std::ostream & | *o* |
|  | ) |  |  |

Definition at line 126 of file generator.hpp.

|  |  |  |  |
| --- | --- | --- | --- |
| void stan::gm::generate\_typedef | ( | const std::string & | *type*, |
|  |  | const std::string & | *abbrev*, |
|  |  | std::ostream & | *o* |
|  | ) |  |  |

Definition at line 310 of file generator.hpp.

|  |  |  |  |  |  |
| --- | --- | --- | --- | --- | --- |
| void stan::gm::generate\_typedefs | ( | std::ostream & | *o* | ) |  |

Definition at line 316 of file generator.hpp.

|  |  |  |  |
| --- | --- | --- | --- |
| void stan::gm::generate\_unconstrained\_param\_names\_method | ( | const program & | *prog*, |
|  |  | std::ostream & | *o* |
|  | ) |  |  |

Definition at line 3491 of file generator.hpp.

|  |  |  |  |
| --- | --- | --- | --- |
| void stan::gm::generate\_using | ( | const std::string & | *type*, |
|  |  | std::ostream & | *o* |
|  | ) |  |  |

Definition at line 288 of file generator.hpp.

|  |  |  |  |
| --- | --- | --- | --- |
| void stan::gm::generate\_using\_namespace | ( | const std::string & | *ns*, |
|  |  | std::ostream & | *o* |
|  | ) |  |  |

Definition at line 292 of file generator.hpp.

|  |  |  |  |  |  |
| --- | --- | --- | --- | --- | --- |
| void stan::gm::generate\_usings | ( | std::ostream & | *o* | ) |  |

Definition at line 297 of file generator.hpp.

|  |  |  |  |
| --- | --- | --- | --- |
| void stan::gm::generate\_validate\_context\_size | ( | std::ostream & | *o*, |
|  |  | const std::string & | *stage*, |
|  |  | const std::string & | *var\_name*, |
|  |  | const std::string & | *base\_type*, |
|  |  | const std::vector< expression > & | *dims*, |
|  |  | const expression & | *type\_arg1* = `expression()`, |
|  |  | const expression & | *type\_arg2* = `expression()` |
|  | ) |  |  |

Definition at line 380 of file generator.hpp.

|  |  |  |  |
| --- | --- | --- | --- |
| void stan::gm::generate\_validate\_positive | ( | const std::string & | *var\_name*, |
|  |  | const expression & | *expr*, |
|  |  | std::ostream & | *o* |
|  | ) |  |  |

Definition at line 458 of file generator.hpp.

|  |  |  |  |
| --- | --- | --- | --- |
| void stan::gm::generate\_validate\_transformed\_params | ( | const std::vector< var\_decl > & | *vs*, |
|  |  | int | *indent*, |
|  |  | std::ostream & | *o* |
|  | ) |  |  |

Definition at line 1504 of file generator.hpp.

|  |  |  |  |
| --- | --- | --- | --- |
| void stan::gm::generate\_validate\_var\_decl | ( | const var\_decl & | *decl*, |
|  |  | int | *indent*, |
|  |  | std::ostream & | *o* |
|  | ) |  |  |

Definition at line 907 of file generator.hpp.

|  |  |  |  |
| --- | --- | --- | --- |
| void stan::gm::generate\_validate\_var\_decls | ( | const std::vector< var\_decl > | *decls*, |
|  |  | int | *indent*, |
|  |  | std::ostream & | *o* |
|  | ) |  |  |

Definition at line 914 of file generator.hpp.

|  |  |  |  |
| --- | --- | --- | --- |
| void stan::gm::generate\_var\_resizing | ( | const std::vector< var\_decl > & | *vs*, |
|  |  | std::ostream & | *o* |
|  | ) |  |  |

Definition at line 537 of file generator.hpp.

|  |  |  |  |  |  |
| --- | --- | --- | --- | --- | --- |
| void stan::gm::generate\_version\_comment | ( | std::ostream & | *o* | ) |  |

Definition at line 334 of file generator.hpp.

|  |  |  |  |
| --- | --- | --- | --- |
| void stan::gm::generate\_void\_statement | ( | const std::string & | *name*, |
|  |  | const size\_t | *indent*, |
|  |  | std::ostream & | *o* |
|  | ) |  |  |

Definition at line 55 of file generator.hpp.

|  |  |  |  |
| --- | --- | --- | --- |
| void stan::gm::generate\_write\_array\_method | ( | const program & | *prog*, |
|  |  | const std::string & | *model\_name*, |
|  |  | std::ostream & | *o* |
|  | ) |  |  |

Definition at line 4131 of file generator.hpp.

|  |  |  |  |
| --- | --- | --- | --- |
| void stan::gm::generate\_write\_csv\_header\_method | ( | const program & | *prog*, |
|  |  | std::ostream & | *o* |
|  | ) |  |  |

Definition at line 3173 of file generator.hpp.

|  |  |  |  |
| --- | --- | --- | --- |
| void stan::gm::generate\_write\_csv\_method | ( | const program & | *prog*, |
|  |  | const std::string & | *model\_name*, |
|  |  | std::ostream & | *o* |
|  | ) |  |  |

Definition at line 3771 of file generator.hpp.

template<typename D >

|  |  |  |  |  |  |
| --- | --- | --- | --- | --- | --- |
| bool stan::gm::has\_lb | ( | const D & | *x* | ) |  |

Definition at line 39 of file generator.hpp.

|  |  |  |  |  |  |
| --- | --- | --- | --- | --- | --- |
| bool stan::gm::has\_lp\_suffix | ( | const std::string & | *s* | ) |  |

Definition at line 1415 of file ast\_def.cpp.

template<typename D >

|  |  |  |  |  |  |
| --- | --- | --- | --- | --- | --- |
| bool stan::gm::has\_lub | ( | const D & | *x* | ) |  |

Definition at line 31 of file generator.hpp.

|  |  |  |  |
| --- | --- | --- | --- |
| bool stan::gm::has\_non\_param\_var | ( | const expression & | *e*, |
|  |  | const variable\_map & | *var\_map* |
|  | ) |  |  |

Definition at line 748 of file ast\_def.cpp.

|  |  |  |  |  |  |
| --- | --- | --- | --- | --- | --- |
| bool stan::gm::has\_only\_int\_args | ( | const function\_decl\_def & | *fun* | ) |  |

Definition at line 4302 of file generator.hpp.

|  |  |  |  |  |  |
| --- | --- | --- | --- | --- | --- |
| bool stan::gm::has\_rng\_suffix | ( | const std::string & | *s* | ) |  |

Definition at line 1406 of file ast\_def.cpp.

template<typename D >

|  |  |  |  |  |  |
| --- | --- | --- | --- | --- | --- |
| bool stan::gm::has\_ub | ( | const D & | *x* | ) |  |

Definition at line 35 of file generator.hpp.

|  |  |  |  |
| --- | --- | --- | --- |
| bool stan::gm::has\_var | ( | const expression & | *e*, |
|  |  | const variable\_map & | *var\_map* |
|  | ) |  |  |

Definition at line 675 of file ast\_def.cpp.

|  |  |  |  |  |  |
| --- | --- | --- | --- | --- | --- |
| const std::string stan::gm::INDENT | ( | " " |  | ) |  |

|  |  |  |  |  |  |
| --- | --- | --- | --- | --- | --- |
| const std::string stan::gm::INDENT2 | ( | " " |  | ) |  |

|  |  |  |  |  |  |
| --- | --- | --- | --- | --- | --- |
| const std::string stan::gm::INDENT3 | ( | " " |  | ) |  |

|  |  |  |  |
| --- | --- | --- | --- |
| expr\_type stan::gm::infer\_type\_indexing | ( | const base\_expr\_type & | *expr\_base\_type*, |
|  |  | size\_t | *num\_expr\_dims*, |
|  |  | size\_t | *num\_index\_dims* |
|  | ) |  |  |

Definition at line 873 of file ast\_def.cpp.

|  |  |  |  |
| --- | --- | --- | --- |
| expr\_type stan::gm::infer\_type\_indexing | ( | const expression & | *expr*, |
|  |  | size\_t | *num\_index\_dims* |
|  | ) |  |  |

Definition at line 892 of file ast\_def.cpp.

|  |  |  |  |
| --- | --- | --- | --- |
| bool stan::gm::is\_assignable | ( | const expr\_type & | *l\_type*, |
|  |  | const expr\_type & | *r\_type*, |
|  |  | const std::string & | *failure\_message*, |
|  |  | std::ostream & | *error\_msgs* |
|  | ) |  |  |

Definition at line 1453 of file ast\_def.cpp.

|  |  |  |  |  |  |
| --- | --- | --- | --- | --- | --- |
| bool stan::gm::is\_linear\_function | ( | const std::string & | *name* | ) |  |

Definition at line 644 of file ast\_def.cpp.

|  |  |  |  |  |  |
| --- | --- | --- | --- | --- | --- |
| bool stan::gm::is\_nil | ( | const expression & | *e* | ) |  |

Definition at line 768 of file ast\_def.cpp.

|  |  |  |  |  |  |
| --- | --- | --- | --- | --- | --- |
| bool stan::gm::is\_nonempty | ( | std::string & | *s* | ) |  |

Definition at line 54 of file parser.hpp.

|  |  |  |  |  |  |
| --- | --- | --- | --- | --- | --- |
| bool stan::gm::is\_space | ( | char | *c* | ) |  |

Definition at line 50 of file parser.hpp.

|  |  |  |  |
| --- | --- | --- | --- |
| bool stan::gm::is\_user\_defined | ( | const std::string & | *name*, |
|  |  | const std::vector< expression > & | *args* |
|  | ) |  |  |

Definition at line 1423 of file ast\_def.cpp.

|  |  |  |  |  |  |
| --- | --- | --- | --- | --- | --- |
| bool stan::gm::is\_user\_defined | ( | const fun & | *fx* | ) |  |

Definition at line 1449 of file ast\_def.cpp.

|  |  |  |  |
| --- | --- | --- | --- |
| bool stan::gm::is\_user\_defined\_prob\_function | ( | const std::string & | *name*, |
|  |  | const expression & | *variate*, |
|  |  | const std::vector< expression > & | *params* |
|  | ) |  |  |

Definition at line 1439 of file ast\_def.cpp.

|  |  |  |  |  |  |
| --- | --- | --- | --- | --- | --- |
| bool stan::gm::needs\_template\_params | ( | const function\_decl\_def & | *fun* | ) |  |

Definition at line 4346 of file generator.hpp.

|  |  |  |  |
| --- | --- | --- | --- |
| std::ostream & stan::gm::operator<< | ( | std::ostream & | *o*, |
|  |  | const expr\_type & | *et* |
|  | ) |  |  |

Definition at line 118 of file ast\_def.cpp.

|  |  |  |  |
| --- | --- | --- | --- |
| bool stan::gm::parse | ( | std::ostream \* | *output\_stream*, |
|  |  | std::istream & | *input*, |
|  |  | const std::string & | *filename*, |
|  |  | const std::string & | *model\_name*, |
|  |  | program & | *result* |
|  | ) |  |  |

inline

Definition at line 61 of file parser.hpp.

|  |  |  |  |
| --- | --- | --- | --- |
| static void stan::gm::print\_quoted\_expression | ( | std::ostream & | *o*, |
|  |  | const expression & | *e* |
|  | ) |  |  |

static

Definition at line 265 of file generator.hpp.

|  |  |  |  |
| --- | --- | --- | --- |
| static void stan::gm::print\_string\_literal | ( | std::ostream & | *o*, |
|  |  | const std::string & | *s* |
|  | ) |  |  |

static

Definition at line 254 of file generator.hpp.

|  |  |  |  |
| --- | --- | --- | --- |
| void stan::gm::print\_var\_origin | ( | std::ostream & | *o*, |
|  |  | const var\_origin & | *vo* |
|  | ) |  |  |

Definition at line 944 of file ast\_def.cpp.

|  |  |  |  |  |  |
| --- | --- | --- | --- | --- | --- |
| expr\_type stan::gm::promote\_primitive | ( | const expr\_type & | *et* | ) |  |

Definition at line 125 of file ast\_def.cpp.

|  |  |  |  |
| --- | --- | --- | --- |
| expr\_type stan::gm::promote\_primitive | ( | const expr\_type & | *et1*, |
|  |  | const expr\_type & | *et2* |
|  | ) |  |  |

Definition at line 131 of file ast\_def.cpp.

|  |  |  |  |
| --- | --- | --- | --- |
| bool stan::gm::returns\_type | ( | const expr\_type & | *return\_type*, |
|  |  | const statement & | *statement*, |
|  |  | std::ostream & | *error\_msgs* |
|  | ) |  |  |

Definition at line 515 of file ast\_def.cpp.

|  |  |  |  |
| --- | --- | --- | --- |
| void stan::gm::suppress\_warning | ( | const std::string & | *indent*, |
|  |  | const std::string & | *var\_name*, |
|  |  | std::ostream & | *o* |
|  | ) |  |  |

Definition at line 2304 of file generator.hpp.

template<typename T >

|  |  |  |  |  |  |
| --- | --- | --- | --- | --- | --- |
| std::string stan::gm::to\_string | ( | T | *i* | ) |  |

Definition at line 44 of file generator.hpp.

|  |  |  |  |  |  |
| --- | --- | --- | --- | --- | --- |
| size\_t stan::gm::total\_dims | ( | const std::vector< std::vector< expression > > & | *dimss* | ) |  |

Definition at line 865 of file ast\_def.cpp.

|  |  |  |  |
| --- | --- | --- | --- |
| std::ostream & stan::gm::write\_base\_expr\_type | ( | std::ostream & | *o*, |
|  |  | base\_expr\_type | *type* |
|  | ) |  |  |

Definition at line 23 of file ast\_def.cpp.

## Variable Documentation

boost::phoenix::function<add\_conditional\_body> stan::gm::add\_conditional\_body\_f

Definition at line 83 of file statement\_2\_grammar\_def.hpp.

boost::phoenix::function<add\_conditional\_condition> stan::gm::add\_conditional\_condition\_f

Definition at line 73 of file statement\_2\_grammar\_def.hpp.

boost::phoenix::function<add\_expression\_dimss> stan::gm::add\_expression\_dimss\_f

Definition at line 574 of file term\_grammar\_def.hpp.

boost::phoenix::function<add\_fun\_var> stan::gm::add\_fun\_var\_f

Definition at line 287 of file functions\_grammar\_def.hpp.

boost::phoenix::function<add\_function\_signature> stan::gm::add\_function\_signature\_f

Definition at line 210 of file functions\_grammar\_def.hpp.

boost::phoenix::function<add\_loop\_identifier> stan::gm::add\_loop\_identifier\_f

Definition at line 460 of file statement\_grammar\_def.hpp.

boost::phoenix::function<add\_while\_body> stan::gm::add\_while\_body\_f

Definition at line 438 of file statement\_grammar\_def.hpp.

boost::phoenix::function<add\_while\_condition> stan::gm::add\_while\_condition\_f

Definition at line 428 of file statement\_grammar\_def.hpp.

boost::phoenix::function<addition\_expr3> stan::gm::addition3\_f

Definition at line 108 of file expression07\_grammar\_def.hpp.

boost::phoenix::function<binary\_op\_expr> stan::gm::binary\_op\_f

Definition at line 102 of file expression\_grammar\_def.hpp.

const int stan::gm::data\_origin = 1

Definition at line 413 of file ast.hpp.

const int stan::gm::derived\_origin = 5

Definition at line 417 of file ast.hpp.

boost::phoenix::function<division\_expr> stan::gm::division\_f

Definition at line 382 of file term\_grammar\_def.hpp.

const int stan::gm::DOUBLE\_T = 2

Definition at line 66 of file ast.hpp.

boost::phoenix::function<elt\_division\_expr> stan::gm::elt\_division\_f

Definition at line 487 of file term\_grammar\_def.hpp.

boost::phoenix::function<elt\_multiplication\_expr> stan::gm::elt\_multiplication\_f

Definition at line 463 of file term\_grammar\_def.hpp.

boost::phoenix::function<exponentiation\_expr> stan::gm::exponentiation\_f

Definition at line 304 of file term\_grammar\_def.hpp.

boost::phoenix::function<expression\_as\_statement> stan::gm::expression\_as\_statement\_f

Definition at line 400 of file statement\_grammar\_def.hpp.

const int stan::gm::function\_argument\_origin = 7

Definition at line 419 of file ast.hpp.

const int stan::gm::function\_argument\_origin\_lp = 8

Definition at line 420 of file ast.hpp.

const int stan::gm::function\_argument\_origin\_rng = 9

Definition at line 421 of file ast.hpp.

const int stan::gm::ILL\_FORMED\_T = 6

Definition at line 70 of file ast.hpp.

const int stan::gm::INT\_T = 1

Definition at line 65 of file ast.hpp.

boost::phoenix::function<left\_division\_expr> stan::gm::left\_division\_f

Definition at line 439 of file term\_grammar\_def.hpp.

const int stan::gm::local\_origin = 6

Definition at line 418 of file ast.hpp.

boost::phoenix::function<logical\_negate\_expr> stan::gm::logical\_negate\_expr\_f

Definition at line 535 of file term\_grammar\_def.hpp.

const int stan::gm::MATRIX\_T = 5

Definition at line 69 of file ast.hpp.

const int stan::gm::model\_name\_origin = 0

Definition at line 412 of file ast.hpp.

boost::phoenix::function<modulus\_expr> stan::gm::modulus\_f

Definition at line 412 of file term\_grammar\_def.hpp.

boost::phoenix::function<multiplication\_expr> stan::gm::multiplication\_f

Definition at line 328 of file term\_grammar\_def.hpp.

boost::phoenix::function<negate\_expr> stan::gm::negate\_expr\_f

Definition at line 514 of file term\_grammar\_def.hpp.

const int stan::gm::parameter\_origin = 3

Definition at line 415 of file ast.hpp.

boost::phoenix::function<remove\_loop\_identifier> stan::gm::remove\_loop\_identifier\_f

Definition at line 470 of file statement\_grammar\_def.hpp.

const int stan::gm::ROW\_VECTOR\_T = 4

Definition at line 68 of file ast.hpp.

boost::phoenix::function<scope\_lp> stan::gm::scope\_lp\_f

Definition at line 242 of file functions\_grammar\_def.hpp.

boost::phoenix::function<set\_allows\_sampling\_origin> stan::gm::set\_allows\_sampling\_origin\_f

Definition at line 109 of file functions\_grammar\_def.hpp.

boost::phoenix::function<set\_fun\_type2> stan::gm::set\_fun\_type2\_f

Definition at line 71 of file expression\_grammar\_def.hpp.

boost::phoenix::function<set\_fun\_type3> stan::gm::set\_fun\_type3\_f

Definition at line 85 of file expression07\_grammar\_def.hpp.

boost::phoenix::function<set\_fun\_type> stan::gm::set\_fun\_type\_f

Definition at line 192 of file term\_grammar\_def.hpp.

boost::phoenix::function<set\_fun\_type\_named> stan::gm::set\_fun\_type\_named\_f

Definition at line 270 of file term\_grammar\_def.hpp.

boost::phoenix::function<set\_var\_type> stan::gm::set\_var\_type\_f

Definition at line 604 of file term\_grammar\_def.hpp.

boost::phoenix::function<set\_void\_function> stan::gm::set\_void\_function\_f

Definition at line 82 of file functions\_grammar\_def.hpp.

boost::phoenix::function<subtraction\_expr3> stan::gm::subtraction3\_f

Definition at line 131 of file expression07\_grammar\_def.hpp.

const int stan::gm::transformed\_data\_origin = 2

Definition at line 414 of file ast.hpp.

const int stan::gm::transformed\_parameter\_origin = 4

Definition at line 416 of file ast.hpp.

boost::phoenix::function<transpose\_expr> stan::gm::transpose\_f

Definition at line 555 of file term\_grammar\_def.hpp.

boost::phoenix::function<unscope\_locals> stan::gm::unscope\_locals\_f

Definition at line 411 of file statement\_grammar\_def.hpp.

boost::phoenix::function<unscope\_variables> stan::gm::unscope\_variables\_f

Definition at line 255 of file functions\_grammar\_def.hpp.

boost::phoenix::function<validate\_allow\_sample> stan::gm::validate\_allow\_sample\_f

Definition at line 508 of file statement\_grammar\_def.hpp.

boost::phoenix::function<validate\_assignment> stan::gm::validate\_assignment\_f

Definition at line 217 of file statement\_grammar\_def.hpp.

boost::phoenix::function<validate\_declarations> stan::gm::validate\_declarations\_f

Definition at line 136 of file functions\_grammar\_def.hpp.

boost::phoenix::function<validate\_expr\_type3> stan::gm::validate\_expr\_type3\_f

Definition at line 67 of file expression07\_grammar\_def.hpp.

boost::phoenix::function<validate\_int\_expr2> stan::gm::validate\_int\_expr2\_f

Definition at line 489 of file statement\_grammar\_def.hpp.

boost::phoenix::function<validate\_int\_expr3> stan::gm::validate\_int\_expr3\_f

Definition at line 620 of file term\_grammar\_def.hpp.

boost::phoenix::function<validate\_integrate\_ode> stan::gm::validate\_integrate\_ode\_f

Definition at line 175 of file term\_grammar\_def.hpp.

boost::phoenix::function<validate\_non\_void\_arg\_function> stan::gm::validate\_non\_void\_arg\_f

Definition at line 62 of file functions\_grammar\_def.hpp.

boost::phoenix::function<validate\_non\_void\_expression> stan::gm::validate\_non\_void\_expression\_f

Definition at line 523 of file statement\_grammar\_def.hpp.

boost::phoenix::function<validate\_return\_allowed> stan::gm::validate\_return\_allowed\_f

Definition at line 102 of file statement\_grammar\_def.hpp.

boost::phoenix::function<validate\_return\_type> stan::gm::validate\_return\_type\_f

Definition at line 233 of file functions\_grammar\_def.hpp.

boost::phoenix::function<validate\_sample> stan::gm::validate\_sample\_f

Definition at line 367 of file statement\_grammar\_def.hpp.

boost::phoenix::function<validate\_void\_return\_allowed> stan::gm::validate\_void\_return\_allowed\_f

Definition at line 121 of file statement\_grammar\_def.hpp.

const int stan::gm::VECTOR\_T = 3

Definition at line 67 of file ast.hpp.

const int stan::gm::void\_function\_argument\_origin = 10

Definition at line 422 of file ast.hpp.

const int stan::gm::void\_function\_argument\_origin\_lp = 11

Definition at line 423 of file ast.hpp.

const int stan::gm::void\_function\_argument\_origin\_rng = 12

Definition at line 424 of file ast.hpp.

const int stan::gm::VOID\_T = 0

Definition at line 64 of file ast.hpp.


---

[ Stan Home Page ]

*© 2011–2014,
Stan Development Team.*
